# Molecular Phylogeny and Infraordinal Classification of Zoraptera (Insecta)

**DOI:** 10.3390/insects11010051

**Published:** 2020-01-12

**Authors:** Petr Kočárek, Ivona Horká, Robin Kundrata

**Affiliations:** 1 Department of Biology and Ecology & Institute of Environmental Technologies, Faculty of Science, University of Ostrava, Chittussiho 10, CZ-710 00 Ostrava, Czech Republic; ivona.horka@osu.cz; 2 Department of Zoology, Faculty of Science, Palacky University, 17. listopadu 50, CZ-771 46 Olomouc, Czech Republic; robin.robyn@centrum.cz

**Keywords:** angel insects, diversity, morphology, evolution, Polyneoptera

## Abstract

Zoraptera is a small and predominantly tropical insect order with an unresolved higher classification due to the extremely uniform external body morphology. We, therefore, conducted a multigene molecular phylogeny of extant Zoraptera and critically re-evaluated their morphological characters in order to propose a natural infraordinal classification. We recovered a highly-resolved phylogeny with two main clades representing major evolutionary lineages in Zoraptera, for which we propose family ranks. The two families exhibit striking differences in male genitalia and reproductive strategies. Each family contains two subclades (subfamilies) supported by several morphological synapomorphies including the relative lengths of the basal antennomeres, the number and position of metatibial spurs, and the structure of male genitalia. The newly proposed higher classification of Zoraptera includes the family Zorotypidae stat. revid. with Zorotypinae Silvestri, 1913 (*Zorotypus* stat. revid., *Usazoros* Kukalova-Peck and Peck, 1993 stat. restit.) and Spermozorinae subfam. nov. (*Spermozoros* gen. nov.), and Spriralizoridae fam. nov. with Spiralizorinae subfam. nov. (*Spiralizoros* gen. nov., *Scapulizoros* gen. nov., *Cordezoros* gen. nov., *Centrozoros* Kukalova-Peck and Peck, 1993, stat. restit., *Brazilozoros* Kukalova-Peck and Peck, 1993, stat. restit.), and Latinozorinae subfam. nov. (*Latinozoros* Kukalova-Peck and Peck, 1993, stat. restit.). An identification key and morphological diagnoses for all supraspecific taxa are provided.

## 1. Introduction

Zoraptera, whose members are commonly known as angel insects, is one of the smallest and least-known insect orders [1,2,3]. This order represents an old evolutionary lineage with a Paleozoic origin [3,4,5]. The systematic placement of this group has always been controversial (see “the Zoraptera problem” introduced by Beutel and Weide [6]), and different insect lineages, such as Psocoptera, Isoptera, and Embioptera, have been proposed as the closest relatives of Zoraptera (see Mashimo et al. [1] for a review). Recent phylogenomic analyses suggest that Zoraptera is a sister group of Dermaptera [3,7,8,9], which had previously been proposed by Terry and Whiting [10].

The higher classification of the angel insects remains unresolved despite the recent research on their morphology and taxonomy [2,5]. Since the order description in 1913 [11], 44 extant species belonging to a single genus, *Zorotypus* Silvestri, 1913, have been described from tropical and subtropical areas of all biogeographical regions [1,12]. Kukalova-Peck and Peck [13] proposed a classification of Zoraptera with seven genera based on wing venation, and Chao and Chen [14] subsequently introduced a new genus, *Formosozoros*, based on an apomorphic species from Taiwan. Engel and Grimaldi [15] critically revised the supraspecific classification of Zoraptera and concluded that the proposed generic characters concerning wing venation are either continuous across taxa or variable within a given species, and that two apomorphies of the Taiwanese species are not generically distinctive. The latter authors, therefore, re-established the single genus *Zorotypus*. Matsumura et al. [5] recently provided the first molecular phylogeny of the order. They identified three main clades, but because unclear morphological support did not allow the classification of *Zorotypus guineensis* Silvestri, 1913 (the type species of a nominotypical genus), they did not propose any changes in classification. Fossil zorapterans are classified partly in *Zorotypus* s. str. (eight species), partly in the exclusively fossil *Zorotypus* subgenus *Octozoros* Engel, 2003 (eight species), which was erected for species with eight antennomeres, and in a monotypic genus *Xenozorotypus* Engel and Grimaldi, 2002 [1,2,16,17]. All of these taxa are currently included in a single family, Zorotypidae.

The body plan of zorapterans is very uniform [1,2], and the majority of external characters are species-specific and do not indicate phylogenetic affinities. The male genitalia seem to be a taxonomically important trait [18,19,20,21], but they are extremely diverse, and homologization of their structures has not yet been implemented [22]. When morphological data are limited in their usefulness for determining the relationships among organisms, a molecular phylogenetic approach can help us to indicate which morphological characters are phylogenetically informative.

In this study, we (a) reconstruct the phylogeny of extant Zoraptera using a combination of nuclear and mitochondrial molecular markers, (b) determine which morphological characters are consistent with the molecular phylogeny, and (c) use the combined molecular and morphological data to propose a robust natural classification of the order.

## 2. Materials and Methods

### 2.1. Taxon Sampling and Laboratory Methods

We sequenced 13 zorapteran specimens representing 12 species from the Neotropical, Panamanian, Madagascan, and Oriental zoogeographic realms (Table 1). Apteron specimens were collected using an aspirator from under the bark of fallen trunks in shady, moist valleys; alates were collected using flight interception traps. We also sequenced seven specimens of Dermaptera (currently considered a sister-group to angel insects [3,4,5,7,8]) representing seven genera in four families (Table 1). Specimens were preserved in 96% ethanol. DNA was extracted from hind legs using the QIAamp DNA Micro Kit (Qiagen, Hilden, Germany) and following the manufacturer’s protocol. We sequenced partial nuclear 18S ribosomal RNA (18S rRNA; usually ~1300 bp), mitochondrial 16S ribosomal RNA (16S rRNA; ~470 bp), and the cytochrome c oxidase subunit I mitochondrial DNA (COI mtDNA; 658 bp), which were used together, in some combinations, or alone in previous molecular studies of Zoraptera [5,23,24] and Dermaptera [25,26,27,28,29]. Polymerase chain reactions (PCR) were performed in 20-μL volumes containing 1 μL of DNA template, 0.4 μM of each primer, 20 mg/mL bovine serum albumin (BSA, New England BioLabs, Ipswich, MA, USA), distilled water, MyTaq Red PCR buffer, and 1 U of MyTaq^TM^ Red DNA polymerase (Bioline Reagents, London, UK). The primers [30,31,32,33,34] and details of PCR conditions are indicated in Table S1. The PCR products were purified using the GenElute PCR Clean-up Kit (Sigma-Aldrich, St. Louis, MO, USA) following the manufacturer’s protocol. Sanger sequencing reactions were performed using a Macrogen ABI3730XL DNA Sequencer (Amsterdam, The Netherlands). The chromatograms were visually checked and manually edited where appropriate using Chromas 2.6.4 (Technelysium, Austria). Voucher specimens are stored as microscopic sections with voucher numbers in the Department of Biology and Ecology at University of Ostrava, Czech Republic. The GenBank accession numbers are indicated in Table 1.

### 2.2. Dataset Assembly and Phylogenetic Analyses

We merged the newly generated data with the available Zoraptera sequences from GenBank [10,23,24]. Our final three-gene dataset comprised 28 taxa, including 21 Zoraptera terminals representing 20 species, and seven species of Dermaptera (Table 1). Multiple sequence alignments of individual genes were constructed using MAFFT 7.157 [35] with default parameters and were subsequently concatenated in Geneious Prime 2020.0.3 [36]. Basic statistics were calculated using MEGA 6.06 [37]. The complete three-gene alignment of 28 taxa contained 2574 homologous positions (18S: 1418 positions; 16S: 498 positions; and COI: 658 positions), including 1306 conserved, 1254 variable, and 1072 parsimony-informative characters. The best-fit model and partitioning schemes were tested using a greedy algorithm in PartitionFinder 1.1.1 [38] under the corrected Akaike information criterion. All phylogenetic analyses were run on the CIPRES web server [39].

The resulting trees were constructed using maximum likelihood analysis (ML) and Bayesian inference (BI). The ML analysis was conducted using RAxML 8.2.9 [40] with the default settings; the data were partitioned by genes and codon positions, and were bootstrapped using rapid Bootstrap algorithm with 1000 pseudoreplicates [41]. For the BI analysis, we used MrBayes 3.2.6 [42] with the GTR+I+G model for most partitions (HKY+I+G for the third COI codon positions) and with partitioning by genes and codon positions as recommended by PartitionFinder. Four chains were run for 40 million generations using the Markov chain Monte Carlo method. Stationary phase and convergence were checked using Tracer 1.7.1 [43]. The first 20% of generations were discarded as burn-in, and the posterior probabilities (PP) were calculated from the remaining trees.

We also evaluated the occurrence of substitution saturation in our data using Xia’s nucleotide substitution saturation test in the software DAMBE 5.6.14 [44,45]. We separately analyzed each non-coding rRNA gene and each position of the protein-coding COI mtDNA using only the fully resolved sites and with the empirical proportion of invariant sites estimated from the data. We detected only a small degree of saturation except for the third codon positions of the COI fragment, which were substantially saturated (Table S2). To test the effect of the saturated third codon positions of the COI mtDNA on the tree topology, we discarded them from the dataset, re-aligned the sequences of individual markers, and re-analyzed the concatenated dataset using both ML and BI analyses with the same settings as described above. All analyzed datasets are available from the corresponding author on request.

### 2.3. Morphology

For observation of morphological and anatomical structures, specimens were soaked in 10% KOH at room temperature for 1 h, washed with distilled water, and returned to 96% ethanol for storage. Observations and dissections were carried out with an Olympus SZX7 stereomicroscope (Olympus Corporation, Tokyo, Japan). Antennae, legs, genitalia, and other body parts were dissected and slide-mounted in Euparal (BioQuip Products, Inc., Compton, CA, USA) and were subsequently observed and documented using an Olympus CX41 microscope equipped with a CANON D1000 camera. Micrographs of 30–150 different focal layers of the same specimen were combined using Quick Photo Camera 2.3 software. All images were edited and assembled into plates in Adobe Photoshop CS6 Extended. We examined the morphology of all Zoraptera specimens that were used for DNA extraction (Table 1) along with the additional material from the following collections: UNFE—Università di Napoli “Federico II”, Napoli, Italy (syntype of *Zorotypus guineensis* Silvestri, 1913; slide mounted legs of *Z. guineensis* from type locality, not labelled as type); BPBM—Bernice Pauahi Bishop Museum, Honolulu, Hawaii, USA (five paratypes of *Z. zimmermani* Gurney, 1939, one specimen of *Z. swezeyi* Caudell, 1922, and 30 specimens of *Z. hubbardi* Caudell, 1918); BMNH - Natural History Museum, London, United Kingdom (holotype of *Z. hamiltoni* New, 1978, two paratypes of *Z. weidneri* New, 1978, and seven specimens of *Z. hubbardi*); ZMH—Zoological Museum Hamburg, Germany (two specimens of *Z. neotropicus* Silvestri, 1916); BYU—Brigham Young University, Provo, UT, USA (two specimens of *Z. novobritannicus* Terry and Whiting, 2012); MMBC—Moravské zemské muzeum, Brno, Czech Republic (two specimens of *Z. delamarei*); UBDC—Institute for Biodiversity and Environmental Research, Universiti Brunei Darussalam, Brunei (two paratypes of *Z. asymmetricus* Kočárek, 2017); and NMPC—National Museum, Prague, Czech Republic (holotype and two paratypes of *Z. asymmetricus*, one specimen of *Z. neotropicus*, and two specimens of *Zorotypus* sp.—*Latinozoros* sp. 1 in our study). Morphological terminology follows Hünefeld [18], Kočárek et al. [46], and Mashimo et al. [12]. Following Engel [47], we used the terms apteron for an individual of the apterous morph, alate for a fully winged individual with developed compound eyes and ocelli, and deälate for an alate that had shed its wings. Classification and definition of world zoogeographic realms follow Holt et al. [48].

## 3. Results

### 3.1. Phylogenetic Analyses

**Figure 1:**
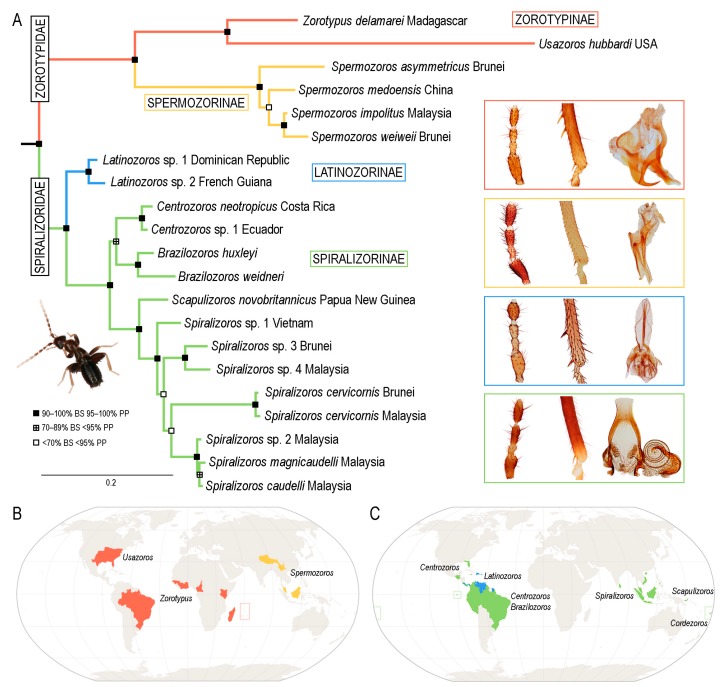
Molecular phylogenetic hypothesis and geographic distribution of Zoraptera. (**A**) Phylogenetic hypothesis for Zoraptera based on the 28 terminals aligned by MAFFT and analyzed by Bayesian inference using MrBayes (outgroup not shown; full tree is in Figure S1). BS = maximum likelihood bootstrap support, PP = Bayesian posterior probabilities. Habitus image represents *Spermozoros asymmetricus* (Kočárek, 2017). Frames on the right include the principal diagnostic characters for all subfamilies (each frame = one subfamily indicated by the same color as in the phylogenetic tree) in the following order (from top frame, left to right): Zorotypinae: basal antennomeres, metatibia and male genitalia of *Zorotypus delamarei* Paulian, 1949, Spermozorinae: basal antennomeres, metatibia and male genitalia of *Spermozoros weiweii* (Wang, Li, and Cai, 2016), comb. nov., Latinozorinae: basal antennomeres, metatibia and male genitalia of *Latinozoros* sp. 1, Spiralizorinae: basal antennomeres of *Spiralizoros cervicornis* (Mashimo, Yoshizawa and Engel, 2013), comb. nov., metatibia of *S. magnicaudelli* (Mashimo, Engel, Dallai, Beutel and Machida, 2013), comb. nov., male genitalia of *S. cervicornis*; (**B**) geographic distribution of Zorotypidae stat. revid.; (**C**) geographic distribution of Spiralizoridae fam. nov. Individual subfamilies in the maps are indicated by different colors as in the phylogenetic tree.

We performed the ML and BI analyses of both the complete dataset and the reduced dataset without the third codon positions of COI. All analyses recovered the same main clades with almost identical (usually 100%) statistical branch-support values (Figure 1A and Figure S1). Zoraptera were divided into two major clades, which we treat as families in this study. The first major clade, i.e., Zorotypidae stat. revid., was further divided into two subclades: the first one contained *Zorotypus delamarei* Paulian, 1949 and *Z. hubbardi*, and the second one contained *Z. medoensis* Huang, 1976, *Z. asymmetricus*, *Z. impolitus* Mashimo, Engel, Dallai, Beutel and Machida, 2013, and *Z. weiweii* Wang, Li and Cai, 2016. The interrelationships within the second subclade differed among analyses. In both ML and BI analyses of the complete dataset, *Z. medoensis* was sister to remaining species, but in the analyses performed on the reduced dataset, *Z. asymmetricus* was either sister to remaining species (BI) or formed a clade with *Z. medoensis*. Neither topology, however, had strong statistical support. *Zorotypus impolitus* and *Z. weiweii* always formed a robustly supported clade. The second major clade showed the same topology in all analyses. The clade, which was formed by two *Zorotypus* spp. from the Dominican Republic and French Guiana, was sister to remaining species. Other moderately to strongly supported basal splits in the second major clade were as follows: (a) *Zorotypus* sp. from Ecuador + *Z. neotropicus* in a clade with *Z. huxleyi* and *Z. weidneri*; (b) *Z. novobritannicus*; and (c) a terminal clade formed by the remaining species. The interrelationships within the terminal clade were not statistically supported even though the same topology was obtained in all analyses. *Zorotypus* sp. from Vietnam was sister to remaining species, and *Z. cervicornis* Mashimo, Yoshizawa and Engel, 2013 (represented by two specimens) was sister to a clade formed by *Z. caudelli* Karny, 1922, *Z. magnicaudelli* Mashimo, Engel, Dallai, Beutel and Machida, 2013 and *Zorotypus* sp. MY1 from Malaysia (Figure 1A and Figure S1).

### 3.2. Infraordinal Classification and Taxonomy

Based on the results of molecular phylogenetic analyses and the detailed re-investigation of morphological characters, we propose here a new classification for Zoraptera, with two families, four subfamilies (two in each family), and nine genera.

#### 3.2.1. Family Zorotypidae Silvestri, 1913, stat. revid.

Type genus. *Zorotypus* Silvestri, 1913, stat. revid.

**Figure 2:**
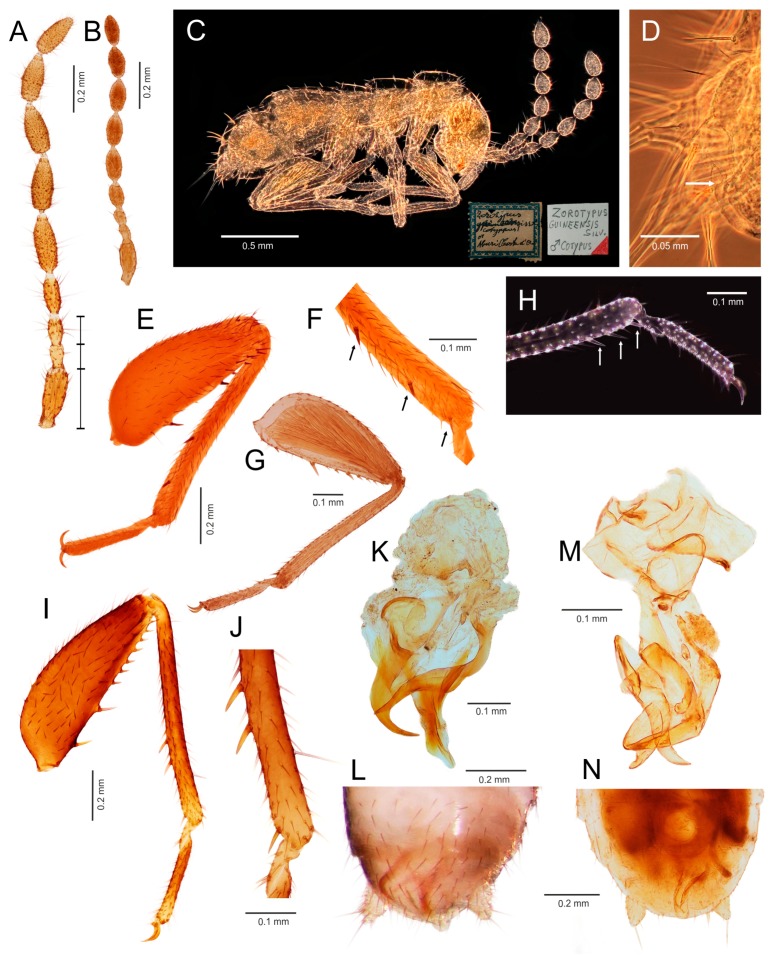
Morphological characters of Zorotypidae: Zorotypinae. (**A**) left antenna of *Zorotypus delamarei* with lengths of the three basal antennomeres indicated, male, Madagascar; (**B**) right antenna of *Usazoros hubbardi*, male, USA; (**C**) slide-mounted type of *Z. guineensis* photographed with phase contrast, with original labels, male, Ghana; (**D**) lateral view of the apex of abdomen of type of *Z. guineensis*, photographed with phase contrast and focused on abdominal tergites, arrow indicate projection of abdominal tergite (probably TXI—see the text), male, Ghana; (**E**) right hind leg of *Z. guineensis*, Ghana, non-type, unknown sex; (**F**) detail of right metatibia of *Z. guineensis*, Ghana, non-type; arrows indicate position of metatibial spurs; (**G**) right hind leg of *U. hubbardi*, male, USA; (**H**) detail of right metatibia of *U. hubbardi* male, USA, photographed with phase contrast and focused only on layer with spurs; arrows indicate position of tibial spurs; (**I**) right hind leg of *Z. delamarei*, male, Madagascar; (**J**) detail of right metatibia of *Z. delamarei*, male, Madagascar; (**K**) genital of *Z. delamarei*, ventral view, male, Madagascar; (**L**) ventral view of the apex of abdomen of *Z. delamarei*, male, Madagascar; (**M**) genitalia of *U. hubbardi*, male, USA; and (**N**) ventral view of the apex of abdomen of *U. hubbardi*, male, USA.

**Figure 3:**
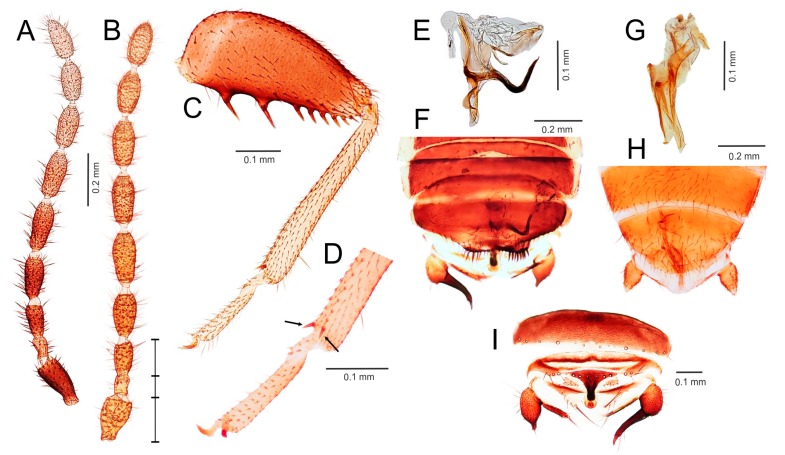
Morphological characters of Zorotypidae: Spermozorinae. (**A**) left antenna of *Spermozoros asymmetricus*, male, Brunei; (**B**) right antenna of *S. weiweii* with lengths of the three basal antennomeres indicated, male, Brunei; (**C**) right hind leg of *S. asymmetricus*, male, Brunei; (**D**) detail of right metatibia of *S. asymmetricus*, male, Brunei; (**E**) male genitalia of *S. asymmetricus*, ventral view, male Brunei; (**F**) ventral view of the apex of male abdomen of *S. asymmetricus*, Brunei; (**G**) male genitalia of *S. weiweii*, ventral view, Brunei; (**H**) ventral view of the apex of male abdomen of *S. weiweii*, Brunei; and (**I**) dorsal view of the apex of male abdomen of *S. asymmetricus*, Brunei.

Diagnosis. Body length 1.5–3.9 mm. Antenna with nine antennomeres, antennomere II short, approximately 0.5–1.0 times as long as antennomere III; antennomeres V–VI short to long, 1.5–3.0 times longer than wide (Figure 2A,B and Figure 3A,B). Metafemur swollen, ventral surface with 5–11 long stout bristles; metatibia with three stout spurs or only two apical spurs (Figure 2H,F,J and Figure 3C,D). Male abdominal tergites X and XI each with a prominent median projection, projection of tergite XI usually extended (mating hook) (Figure 3I). Cercus unsegmented, conical, 1.0–2.5 times as long as wide (Figure 2L,N and Figure 3F,H,I). Male genitalia asymmetrical (Figure 2K,M and Figure 3E,G), composed of two to three claspers, which are prominent posteriorly. Aedeagus conical, posteroventrally pointed. Elongate intromittent organ and basal plate not developed.

Taxa included. 14 described extant species classified in four genera and two subfamilies.

Distribution. Afrotropical, Madagascan, Neotropical, Nearctic, and Oriental regions (Figure 1B).

##### Subfamily Zorotypinae Silvestri, 1913

ZooBank: http://zoobank.org/urn:lsid:zoobank.org:act:37B7FCBF-1D20-47E7-B06F-A6C18694149B

Type genus. *Zorotypus* Silvestri, 1913, stat. revid.

Diagnosis. Body length 1.5–3.0 mm. Antenna with nine antennomeres, antennomere I long, 2.5–3.0 times longer than antennomere III, antennomere II short, approximately as long as antennomere III; antennomeres V–VI short to long, 1.5–3.0 times longer than wide (Figure 2A,B). Pronotum subrectangular, as wide as long or slightly wider than long. Metafemur swollen, ventral surface with 5–11 long, stout bristles (Figure 2E,G,I); metatibia with three stout or weak spurs, only one of which is located apically (Figure 2H,F,J). Male abdominal tergites X and XI each with a prominent median projection, projection of tergite XI usually extended (mating hook). Male sternite VIII with apex concave or with one or two medial protuberances. Cercus unsegmented, conical, 1.0–2.5 times as long as wide (Figure 2L,N). Male genitalia asymmetrical (Figure 2K,M), with two prominent claspers posteriorly (aedeagus and outer paramere). Aedeagus conical, posteroventrally pointed; left outer paramere ventrally or posteroventrally hooked and pointed. Elongate intromittent organ and basal plate not developed.

Genera included. *Zorotypus* Silvestri, 1913, stat. revid.; *Usazoros* Kukalova-Peck and Peck, 1993, stat. restit.

Distribution. Afrotropical, Madagascan, Neotropical, and Nearctic regions (Figure 1B).

###### Genus *Zorotypus* Silvestri, 1913, stat. revid.

Type species. *Zorotypus guineensis* Silvestri, 1913.

Diagnosis. Body length 1.5–3.0 mm, basic color from ocher to dark brown. Antenna with nine antennomeres, antennomere I long, three times longer than antennomere III, antennomere II short, approximately as long as antennomere III; antennomeres V–VI short to long, 1.5–2.5 times longer than wide (Figure 2A,C). Pronotum subrectangular, as wide as long or slightly wider than long. Metafemur swollen, ventral surface with 5–14 long bristles (Figure 2E,I); metatibia with three stout spurs, only one of which is located apically (Figure 2F,J). Male abdominal tergite X membranous, with sclerotized median and lateral parts, medially narrowed, with spatula-like projection, tergite XI with extended median projection (mating hook). Apex of male abdominal sternum VIII concave or with one/two median protuberances. Cercus unsegmented, conical, as long as wide to slightly longer than wide (Figure 2L). Male genitalia asymmetrical (Figure 2K), ventral sac-like structure (pouch of endophallus) enclosed by small asymmetrical sclerites. Aedeagus conical, posteroventrally pointed; left outer paramere ventrally or posteroventrally hooked and pointed. Elongate intromittent organ and basal plate not developed.

Species included. *Zorotypus guineensis* Silvestri, 1913; *Z. asymmetristernum* Mashimo, 2019; *Z. delamarei* Paulian, 1949; *Z. vinsoni* Paulian 1951; *Z. shannoni* Gurney, 1938; *Z. amazonensis* Rafael and Engel, 2006; and *Z. caxiuana* Rafael, Godoi and Engel, 2008.

Distribution. Afrotropical region: Kenya, Guinea, Ghana, and Ivory Coast; Madagascan region: Madagascar, Mauritius; Neotropical region: Brazil (Figure 1B).

Comments: The type specimen of *Z. guineensis* and all other known material from Silvestri’s collection are slide-mounted and are therefore unavailable for DNA extraction (Figure 2C). Dallai et al. [21] studied Silvestri’s slides, and although the aedeagus and other details could not be observed, the authors were able to prove the absence of a coiled intromittent organ. From the shape and position of body it is evident, that Figures II and VIII in Silvestri [11] are line drawings based on the here illustrated type (Figure 2C). Since not all structures and especially the external genitalia are visible on the slide, we can assume that the drawing is a consensus of observations of more specimens. The lateral and compressed position of the male type specimen complicates the observation of the apical portion of abdomen which is covered by cerci. Presence of the abdominal projections is documented by a trace of projection on one of the abdominal tergites (Figure 2D). Due to its position between the bases of cerci, it is probably a projection of the tergite XI. Although the structure of *Z. guineensis* external genitalia is not known, all other characters used in the genus diagnosis (number of metatibial spurs, arrangement of basal antennomeres, presence of at least one small projection on abdominal tergite X or XI) agree with those of *Z. delamarei*, which was used in the molecular phylogeny (Figure 2A,I–L). Therefore, we are confident that these species belong to the same (nominotypical) genus.

Matsumura et al. [5] used different species of *Zorotypus* stat. restit. in their phylogenetic analyses and obtained different topology with clades *Usazoros hubbardi*/*Spermozoros impolitus* and *Zorotypus asymmetristernum*/*Z*. *shannoni*/*Zorotypus* sp.1 Cameroon in a sister position. *Z. delamarei* used in our study is morphologically similar (and supposedly closely related) to *Z. asymmetristernum* used by Matsumura et al. [5]. *Zorotypus*, as defined here, is broadly defined and includes species sharing morphological characters on genitalia, metatibia, antennae, and abdominal tergites. However, we cannot exclude that some lineages of the currently defined *Zorotypus* will receive a generic status after more extensive morphological and molecular analyses. Neotropical *Zorotypus shannoni*, *Z. caxiuana*, and *Z. amazonensis* share morphological diagnostic characters of *Zorotypus*, especially the structure of male genitalia and the arrangement of spurs on metatibia [50,51,52]. Rafael et al. [53] introduced a “*shannoni*” species group for the three above-mentioned species. They considered a hairy patch on the vertex as a diagnostic character of this group, although such setae are absent in *Z. shannoni* [5]. Some other *Zorotypus* species (e.g., *Z. asymmetristernum*) have a hairy patch on the vertex; the patch is probably associated with the cephalic gland described in *Latinozoros barberi*, and a similar structure is also present in unrelated *Spermozoros weiweii* and *S. impolitus.* The placement of the “*shannoni*” species group in the newly circumscribed *Zorotypus* is supported also by the molecular phylogenetic analysis by Matsumura et al. [5], but further analyses are necessary to underpin the proposed classification.

###### *Usazoros* Kukalova-Peck and Peck, 1993, stat. restit.

Type species. *Zorotypus hubbardi* Caudell, 1918; designated by Kukalova-Peck and Peck [13].

Diagnosis. Body length 1.8–2.4 mm, basic color from ocher to light brown. Antenna with nine antennomeres, antennomere I long, two times longer than antennomere III, antennomere II short, approximately as long as antennomere III; antennomeres V–VI moderately long, two times longer than wide (Figure 2B). Male vertex without hairy patch. Pronotum subrectangular, as wide as long, slightly narrowed posteriorly. Metafemur more swollen proximally than distally, ventral surface with 12–14 more or less stout bristles (Figure 2G); metatibia with three weakly developed spurs in distal fourth, of which one is located apically (Figure 2H). Male abdominal tergites X and XI without prominent hook, with only weak median protuberance. Apex of male abdominal sternite VIII rounded. Cercus unsegmented, conical, approximately two times longer than wide (Figure 2N). Male genitalia asymmetrical (Figure 2M), asymmetrical aedeagus with apical fold with hooked apex. Left outer paramere elongated, with slightly curved apex [18,19]. Endophallus reinforced by several small sclerites arranged in asymmetric manner [18]. Elongate intromittent organ and basal plate not developed.

Species included. *Usazoros hubbardi* (Caudell, 1918), stat. restit.

Distribution. Nearctic region: USA (Figure 1B).

Comments. This genus was described by Kukalova-Peck and Peck [13] exclusively based on wing venation. Engel and Grimaldi [15] synonymized this genus with *Zorotypus* Silvestri, 1913 due to the variability in characters used for the generic diagnosis. Here, we reinstate the name *Usazoros* with a new diagnosis based on several morphological characters excluding wing venation.

##### Subfamily Spermozorinae subfam. nov.

ZooBank: http://zoobank.org/urn:lsid:zoobank.org:act:D40BA7C4-C243-40B9-9924-9610815F2C61

Type genus. *Spermozoros* gen. nov.

Diagnosis. The same as for the genus *Spermozoros* gen. nov. (see below).

Genus included. *Spermozoros* gen. nov.

Distribution. Oriental region (Figure 1B).

Etymology. Derived from the genus *Spermozoros* gen. nov.

###### Genus *Spermozoros* gen. nov.

ZooBank: http://zoobank.org/urn:lsid:zoobank.org:act:3EDFD566-2EFE-473E-A3B7-EC328D6B9AD2

Type species. *Zorotypus asymmetricus* Kočárek, 2017; here designated.

Diagnosis. Body length 2.0–3.9 mm, basic color dark brown. Antenna with nine antennomeres, antennomere I approximately 1.3 times longer than antennomere III, antennomere II slightly curved, short, about 0.5 times as long as antennomere III; antennomeres V–VI long, 2.5–3.0 times longer than wide (Figure 3A,B). Vertex with hairy patch (*S. weiweii*, *S. impolitus,* and *S. huangi*), or without (*S. asymmetricus*, *S. medoensis*, and *S. sinensis*). Pronotum subrectangular, longer than wide or as wide as long, slightly narrowed posteriorly. Metafemur more swollen proximally than distally, ventral surface with 6–11 long stout bristles (Figure 3C); metatibia with two apical spurs (Figure 3D). Male abdominal tergite X with spatula-like median projection, tergite XI strongly curved upward (Figure 3I). Apex of male abdominal sternite VIII usually asymmetrical, with one or two tubercles; when tubercles absent, then sternite concave medially. Male genitalia asymmetrical (Figure 3E–H), composed of three sclerites, middle sclerite spatula-like. Aedeagus hooked or straight (Figure 3E,G). Elongate intromittent organ and basal plate not developed.

Species included. *Spermozoros asymmetricus* (Kočárek, 2017), comb. nov.; *S. medoensis* (Huang, 1976), comb. nov.; *S. weiweii* (Wang, Li and Cai, 2016), comb. nov.; *S. impolitus* (Mashimo, Engel, Dallai, Beutel and Machida 2013), comb. nov.; *S. huangi* (Yin and Li, 2017), comb. nov.; and *S. sinensis* (Huang, 1974), comb. nov.

Distribution. Oriental region: China, Malaysia, Indonesia (Kalimantan) (Figure 1B).

Etymology. *Spermozoros impolitus* produces a single, giant sperm in a spermatophore, and then attaches the spermatophore to the female postabdomen through an external sperm transfer [20]. The generic name refers to this fact in combination with a suffix derived from the word base of the order name. Gender masculine.

Comments. The genus is composed of species with two types of male genitalia: type A has a hooked and strongly sclerotized aedeagus (Figure 3E,F), and type B is composed of three small, straight, and weakly sclerotized sclerites (Figure 3G,H). Type A genitalia are characteristic of *S. asymmetricus*, *S. medoensis*, and *S. sinensis*, while type B genitalia are present in *S. weiweii*, *S. impolitus*, and *S. huangi* [54,55,56,57].

#### 3.2.2. Family Spiralizoridae fam. nov.

ZooBank: http://zoobank.org/urn:lsid:zoobank.org:act:D17B565E-67BA-486A-B7B0-EC8887907277

Type genus. *Spiralizoros* gen. nov.

**Figure 4:**
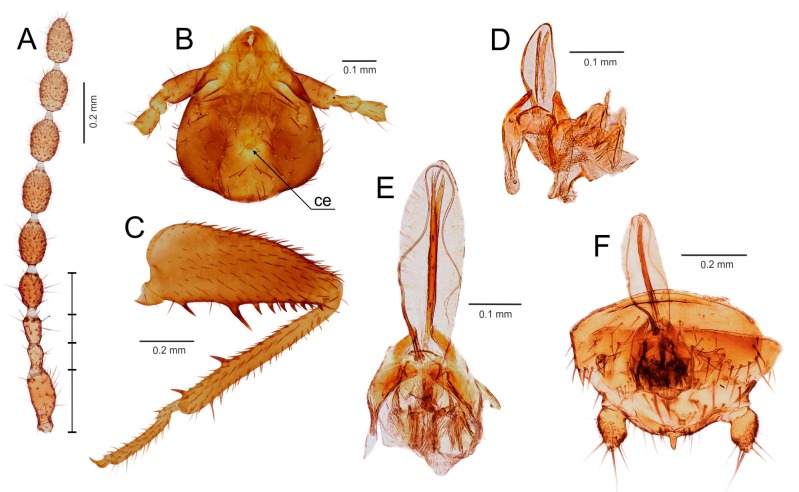
Morphological characters of Spiralizoridae: Latinozorinae. (**A**) right antenna of *Latinozoros* sp. 1 with lengths of the three basal antennomeres indicated, male, Dominican Republic; (**B**) dorsal view of head of *Latinozoros* sp. 1, male, Dominican Republic; ce - cephalic gland; (**C**) right hind leg of *Latinozoros* sp. 1, male, Dominican Republic; (**D**) male genitalia of *Latinozoros* sp. 2, latero-ventral view, French Guiana; (**E**) male genitalia of *Latinozoros* sp. 1, ventral view, Dominican Republic; (**F**) abdomen end of *Latinozoros* sp. 1, male, Dominican Republic.

**Figure 5:**
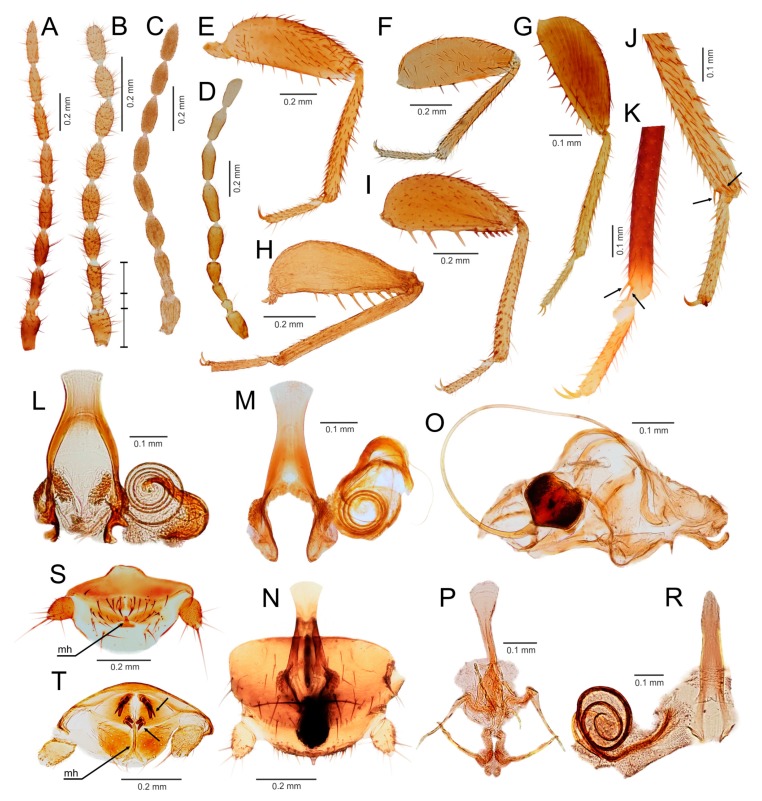
Morphological characters of Spiralizoridae: Spiralizorinae. (**A**) right antenna of *Spiralizoros cervicornis*, male, Brunei; (**B**) left antenna of *Spiralizoros magnicaudelli* with lengths of the three basal antennomeres indicated, male, Malaysia; (**C**) right antenna of *Scapulizoros novobritannicus*, female, Papua New Guinea (New Britain); (**D**) right antenna of *Brazilozoros weidneri*, male, Brazil; (**E**) right hind leg of *Spiralizoros cervicornis*, male, Brunei; (**F**) right hind leg of *Brazilozoros weidneri*, male, Brazil; (**G**) right hind leg of *Centrozoros* sp. 1, female, Ecuador; (**H**) right hind leg of *Scapulizoros novobritannicus*, female, Papua New Guinea (New Britain); (**I**) right hind leg of *Cordezoros zimmermani*, female, Fiji; (**J**) detail of right metatibia of *Centrozoros neotropicus*, female, Costa Rica; arrows indicate apical spurs (**K**) detail of right metatibia of *Spiralizoros magnicaudelli*, male, Malaysia; arrows indicate apical spurs (**L**) male genitalia of *Spiralizoros cervicornis*, Brunei; (**M**) male genitalia of *Spiralizoros magnicaudelli*, ventral view, Malaysia; (**N**) ventral view of the apex of abdomen of *Spiralizoros magnicaudelli*, male, Malaysia; (**O**) male genitalia of *Cordezoros zimmermani*, lateral view, Fiji; note: symmetry of genitalia and position of a heart-like sclerite are shifted due to slide mounting; (**P**) male genitalia of *Brazilozoros weidneri*, ventral view, Brazil; (**R**) male genitalia of *Centrozoros hamiltoni*, ventral view, Colombia; (**S**) abdomen end of *Spiralizoros magnicaudelli* in caudal view, male, Malaysia; mh—mating hook on tergite XI; (**T**) abdominal apex of *Brazilozoros weidneri*, male, Brazil; arrows indicate sensilla, mh - mating hook on tergite XI.

Diagnosis. Body length 1.9–3.6 mm, basic color from pale yellow/light brown to dark brown, alates and deälates darker. Antenna with nine antennomeres (Figure 4A and Figure 5A–D). Pronotum quadratic or trapezoid, as wide as long or slightly wider than long. Metafemur swollen, ventral surface with 3–10 long bristles; metatibia with two stout spurs (first one in distal third, second one near apex), with only two apical spurs, or without spurs (Figure 4C and Figure 5E–K). Male abdominal tergite X partly membranous, with or without median projection, tergite XI with median upcurved projection (mating hook) (Figure 4F and Figure 5S,T); apex of male abdominal sternite VIII rounded, medially incised or with projection. Male genitalia symmetrical (Figure 4D–F and Figure 5L–R), intromittent organ usually developed, sclerotized, and coiled.

Taxa included. Twenty-two described extant species classified in six genera and two subfamilies.

Distribution. Oriental, Nearctic, Panamanian, Neotropical, and Oceanian regions (Figure 1C).

##### Subfamily Latinozorinae subfam. nov.

ZooBank: http://zoobank.org/urn:lsid:zoobank.org:act:7222D11C-2D9A-464A-8451-DC1AC68CDD81

Type genus. *Latinozoros* Kukalova-Peck and Peck, 1993, stat. restit.

Diagnosis. The same as for the genus *Latinozoros* gen. nov. (see below).

Genus included. *Latinozoros* Kukalova-Peck and Peck, 1993, stat. restit

Distribution. Panamanian and Neotropical regions (Figure 1C).

Etymology. Derived from the genus name *Latinozoros* gen. nov. 

###### Genus *Latinozoros* Kukalova-Peck and Peck, 1993, stat. restit.

Type species. *Zorotypus barberi* Gurney, 1938; designated by Kukalova-Peck and Peck [13].

Diagnosis. Body length 1.9–2.3 mm, basic color of apterons from ocher to light brown, alates and deälates darker. Antenna with nine antennomeres, antennomere I long, 3.0 times longer than antennomere III, II antennomere short, slightly shorter than antennomere III; antennomeres V–VI short, 1.5–1.7 times longer than wide (Figure 4A). Small cephalic gland presents in the centre of vertex (Figure 4B). Pronotum subrectangular, slightly wider than long. Metafemur swollen basally, ventral surface with 8–9 long bristles; metatibia with two stout spurs, one of which is located apically (Figure 4C). Male abdominal tergite X with spatula-like projection, tergite XI with hooked median projection (Figure 4F). Apex of male abdominal sternite VIII slightly concave. Cercus unsegmented, conical, two times longer than wide (Figure 4F). Male genitalia symmetrical (Figure 4D–F), composed of a weakly sclerotized (membranous) anterior projection, a pair of dorsal lobes, a pair of mesal lobes, and a long, needle-like aedeagus between mesal lobes. Intromittent organ long, encircling membranous projection (Figure 4E).

Species included. *Latinozoros barberi* (Gurney, 1938), stat. restit.; *Latinozoros* sp. 1; and *Latinozoros* sp. 2.

Distribution. Panamanian region: Panama, Costa Rica, Venezuela, Dominican Republic, and Trinidad; Neotropical region: French Guiana (Figure 1C).

Comments. This genus was described by Kukalova-Peck and Peck [13] based exclusively on wing venation characters. Engel and Grimaldi [15] revised the diagnoses and synonymized this genus with *Zorotypus* Silvestri, 1913 because the described generic characters are variable within a given species and continuous across taxa. According to the Principle of Priority (ICZN: Articles 23.1 and 23.3 of the Code), *Latinozoros* Kukalova-Peck and Peck, 1993 is the oldest available name for this taxon. Therefore, we reinstated this name with a new diagnosis based on a set of morphological characters.

The male genitalia differ between populations from Dominican Republic and French Guiana, and both populations differ significantly in male genitalia from the holotype from the Cocos Island [50]. The antennae, hind legs, and abdominal apex, however, are uniform among the examined populations of this species. The uncorrected pairwise genetic distance between *Latinozoros* sp. 1 and *Latinozoros* sp. 2 based on the COI gene fragment is 18.1%. As currently defined, *Latinozoros barberi* appears to be a species complex, and examination of populations from different areas is clearly needed.

##### Subfamily Spiralizorinae subfam. nov.

ZooBank: http://zoobank.org/urn:lsid:zoobank.org:act:3E79AA25-91DF-4BFD-9C58-848383841A5A

Type genus. *Spiralizoros* gen. nov.

Diagnosis. Body length 2.0–3.6 mm, basic color from pale yellow/light brown to dark brown, alates and deälates darker. Antenna with nine antennomeres, antennomere I approximately 0.7–1.0 times as long as antennomere III, antennomere III about 1.5–2.0 times longer than antennomere II; antennomeres V–VI 2.0–3.0 times longer than wide (Figure 5A–D). Pronotum quadratic or trapezoid, as wide as long to wider than long. Metafemur swollen, ventral surface with 3–10 long moderately stout bristles; metatibia with two apical spurs or without spurs (Figure 5E–K). Male abdominal tergite X partly membranous, without median projection, tergite XI with median upcurved projection (mating hook) (Figure 5S,T); apex of male abdominal sternite VIII rounded, medially incised or with projection. Male genitalia symmetrical (Figure 5L–R), basal plate enlarged and well sclerotized, posteriorly bifurcate, with anterior tongue-like process; elongated intromittent organ usually developed, sclerotized, and coiled.

Genera included. *Spiralizoros* gen. nov., *Centrozoros* Kukalova-Peck and Peck, 1993, stat. restit., *Cordezoros* gen. nov., *Scapulizoros* gen. nov., and *Brazilozoros* Kukalova-Peck and Peck, 1993, stat. restit.

Distribution. Oriental, Nearctic, Panamanian, Neotropical, and Oceanian regions (Figure 1C).

Etymology. Derived from the genus *Spiralizoros* gen. nov.

###### Genus *Spiralizoros* gen. nov.

ZooBank: http://zoobank.org/urn:lsid:zoobank.org:act:98F58F83-368D-46C4-9F85-41D71C97C427

Type species. *Zorotypus cervicornis* Mashimo, Yoshizawa and Engel, 2013; here designated.

Diagnosis. Body length 2.0–3.5 mm, basic color of apteron specimens pale yellow/light brown, alates and deälates darker. Antenna with nine antennomeres, antennomere I short, 1.0–1.3 times as long as antennomere III, antennomere II slightly curved, short, about half the length of antennomere III; antennomeres V–VI 2.0–2.5 times longer than wide (Figure 5A,B). Pronotum quadratic, as wide as long or slightly wider than long. Metafemur are more swollen proximally than distally; ventral surface with 4–10 long moderately stout bristles (Figure 5E); metatibia with two apical spurs (Figure 5K). Male abdominal tergite X posteromedially incised, without median projection; tergite XI with small median upcurved projection (mating hook) (Figure 5S); apex of male abdominal sternite VIII rounded. Male genitalia symmetrical (Figure 5L–N), basal plate enlarged and well sclerotized, posteriorly bifurcate, with anterior tongue-like process, which is dilated anteriorly with diverging sides; intromittent organ long, sclerotized, and coiled; aedagus with hook (Figure 5L,M).

Species included. *Spiralizoros cervicornis* (Mashimo, Yoshizawa and Engel, 2013), comb. nov.; *S. caudelli* (Karny, 1922), comb. nov.; *S. hainanensis* (Yin, Li, and Wu, 2015), comb. nov.; *S. magnicaudelli* (Mashimo, Engel, Dallai, Beutel and Machida, 2013), comb. nov.; *S. silvestrii* (Karny, 1927), comb. nov.; *S. ceylonicus* (Silvestri, 1913), comb. nov.; *S. philippinensis* (Gurney, 1938), comb. nov.; *S. buxtoni* (Karny, 1932), comb. nov.; *Spiralizoros* sp. 1 (Vietnam) [23]; *Spiralizoros* sp. 2 (Malaysia) [23]; *Spiralizoros* sp. 3 (Brunei); and *Spiralizoros* sp. 4 (Borneo).

Distribution. Oriental region: China, Vietnam, Malaysia, Indonesia (Kalimantan, Sumatra, Java), Sri Lanka, and Philippines (Figure 1C).

Etymology. The generic name refers to the coiled (Latin word: spiralis) intromittent organ, which is a typical character shared by all members of this genus, in combination with a suffix derived from the word base of the order name. Gender masculine.

Comments. This genus contains species with a coiled intromittent organ and an enlarged basal plate with an anterior tongue-like process, which is dilated anteriorly with diverging sides. We selected *Zorotypus cervicornis* as the type species of *Spiralizoros* gen. nov., although *S. caudelli* is the oldest described species in this genus. The reason is the taxonomical instability of *S. caudelli*, which seems to be a complex of morphologically similar species. This species was originally described from Southern Sumatra by Karny [58] and was later reported also from Borneo [59] and Peninsular Malaysia [55]. The original description [60] is not detailed, and the type material has probably been lost [59]. Mashimo et al. [55] redescribed *S. caudelli* based on material from Peninsular Malaysia, but without designating a neotype. The specimens from Borneo examined and illustrated by New [59] probably represent an undescribed species. The population from Peninsular Malaysia that was used for the redescription of *S. caudelli* by Mashimo et al. [55] may represent yet another species, because the original drawings of *S. caudelli* from Sumatra by Karny [60] do not fully agree in all characters with *S. caudelli* from Malaysia (especially the shape of cerci and the metafemur spination). Rigorous re-evaluation of the taxonomic status of *S. caudelli* is therefore needed. Information concerning *Spiralizoros* sp. 2 from Peninsular Malaysia is limited to the DNA sequence published by Yoshizawa and Johnson [23]. Mashimo et al. [55] considered that sequence belonging to *S. caudelli*, but we found differences in that sequence and those from specimens collected at the locality from which the authors used the material for redescription.

*Spiralizoros ceylonicus*, *S. silvestrii*, *S. buxtoni*, and *S. philippinensis* are known only from the type specimens, which seem to be lost (*S. ceylonicus*, *S. silvestrii*, and *S. buxtoni*), or which were described based only on females (*S. philippinensis*). Description and illustrations of these species fit well with the characters typical for *Spiralizoros* as diagnosed here (although male genitalia are not illustrated in any case), including the basal antennomere lengths, pronotum wider than long, and pattern of the metafemur spination [11,50,58,61]. Determining the validity and relationships of these species will require further examination, ideally using material collected at type localities*. Spiralizoros buxtoni* is known only from a single female type, which has elongated cerci that terminate in a thick seta and which, therefore, resemble the cerci of *S. ceylonicus* and *S. silvestrii*.

###### *Centrozoros* Kukalova-Peck and Peck, 1993, stat. restit.

*Meridozoros* Kukalova-Peck and Peck, 1993 (type species: *Zorotypus leleupi* Weidner, 1976); synonymized by Engel and Grimaldi [15].

*Floridazoros* Kukalova-Peck and Peck, 1993 (type species: *Zorotypus snyderi* Caudell, 1920); synonymized by Engel and Grimaldi [15].

Type species. *Zorotypus gurneyi* Choe, 1989; designated by Kukalova-Peck and Peck [13].

Diagnosis. Body length 2.3–3.6 mm, basic color of apteron specimens are pale yellow/light brown; alates and deälates are darker. Antennae with nine antennomeres, antennomere I 1.0–1.5 times longer than antennomere III, antennomere II short, about one-half the length of antennomere III; antennomeres V–VI approximately 2.5 times longer than wide. Pronotum quadratic, slightly longer or shorter than wide, or as wide as long. Metafemur swollen; ventral surface with 6–10 long moderately stout bristles (Figure 5G); metatibia with doubled apical spur (Figure 5J) or spurs completely missing. Male tergite X partly membranous, without median projection; tergite XI with median upcurved projection (mating hook); apex of male abdominal sternite VIII rounded, medially incised or with projection. Male genitalia symmetrical (Figure 5R), basal plate enlarged and well sclerotized, posteriorly bifurcate, with anterior tongue-like process, which has converging edges and is never dilated anteriorly; intromittent organ sclerotized and coiled; aedagus with hook.

Species included. *Centrozoros snyderi* (Caudell, 1920), stat. restit.; *C. cramptoni* (Gurney, 1938); *C. gurneyi* (Choe, 1989); *C. hamiltoni* (New, 1978); *C. mexicanus* (Bolívar y Pieltain, 1940); *C. neotropicus* (Silvestri, 1916); *C. manni* (Caudell, 1923), and *Centrozoros* sp. 1 (unpublished).

Distribution. Nearctic region: USA; Panamanian region: Jamaica, Guatemala, Costa Rica, Panama, and Mexico; Neotropical region: Colombia, Brazil, Ecuador, and Peru (Figure 1C).

Comments. This genus was described by Kukalova-Peck and Peck [13] based only on the wing venation of *Centrozoros gurneyi* (Choe, 1989). Engel and Grimaldi [15] synonymized it with *Zorotypus* Silvestri, 1913 based on the variability of characters used for the generic diagnosis. According to the Principle of Priority (ICZN 1999; Articles 23.1 and 23.3 of the Code), *Floridazoros* Kukalova-Peck and Peck, 1993, *Meridozoros* Kukalova-Peck and Peck, 1993, and *Centrozoros* Kukalova-Peck and Peck, 1993 are available names published in the same study. Among these names, we selected *Centrozoros* Kukalova-Peck and Peck, 1993 because it is taxonomically well described and illustrated [5,62,63].

Only two species of *Centrozoros* were included in our analyses, and the remaining species were assigned to this genus based on the morphology of antennae, hind legs, abdomen, and male genitalia. Additional research is therefore needed on the monophyly of this genus.

*Centrozoros* includes species with an enlarged basal plate with a coiled intromittent organ and an anterior tongue-like process that is not dilated anteriorly. *Centrozoros manni* is described based on a single dealate female [64], and only females have been recorded to date [5]. Although this species cannot be classified to a genus based on the morphological description, the molecular analysis of Matsumura et al. [5] clearly suggests its position in *Centrozoros*.

###### *Brazilozoros* Kukalova-Peck and Peck, 1993, stat. restit.

Type species. *Zorotypus brasiliensis* Silvestri, 1946, designated by Kukalova-Peck and Peck [13].

Diagnosis. Body length 2.2–2.7 mm, basic color light to dark brown. Antenna with 9 antennomeres, antennomere I as long as antennomere III, antennomere III about 1.5 times longer than antennomere II; antennomeres V–VI approximately 2.0–2.5 times longer than wide (Figure 5D). Pronotum trapezoid, as wide as long, narrowed posteriorly. Metafemur swollen; ventral surface with 6–15 long weak to moderately stout bristles; metatibia with two apical spurs (Figure 5E). Male abdominal tergite X membranous medially, without median projection; tergite XI with small or long upcurved projection (mating hook) (Figure 5T). Tergite X (XI) with thickened black basiconic sensilla (Figure 5T); apex of male abdominal sternite VIII rounded, broadly emarginated posteriorly. Male genitalia symmetrical (Figure 5P), basal plate enlarged and well sclerotized, with anterior tongue-like process; elongated intromittent organ not developed.

Species included. *Brazilozoros brasiliensis* (Silvestri, 1947), comb. nov., *Brazilozoros weidneri* (New, 1978), comb. nov., and *Brazilozoros huxleyi* (Bolívar y Pieltain and Coronado, 1963), comb. nov.

Distribution. Neotropical region: Brazil, Peru, Guyana, and Ecuador (Figure 1C).

Comments. This genus includes three species, two of which have an enlarged basal plate of the male genitalia but which lack an elongated intromittent organ [5,51,65]; the third species (*B. brasiliensis*) is known only from females [51,52,53,66] and may, therefore, be parthenogenetic [51,66]. The classification of these three species to a single genus is supported by the results of Matsumura et al. [5]. The enlarged basal plate and hind tibiae with only a pair of apical spurs support the assignment of this genus to the Spiralizorinae. Males of *B. weidneri* have typical thick sensilla on abdominal tergite X, and males of *B. huxleyi* have similar sensilla on tergite X and XI [5,65].

###### *Cordezoros* gen. nov.

ZooBank: http://zoobank.org/urn:lsid:zoobank.org:act:4718745E-6453-40B6-A02A-A61DBAF4DA9A

Type species. *Zorotypus zimmermani* Gurney, 1939; here designated.

Diagnosis. Body length 2.1–2.4 mm, basic color of apteron and alates/deälates pale brown. Antenna with nine antennomeres, antennomere I short, up to 2/3 the length of antennomere III, antennomere III 2.0 times longer than antennomere II; antennomeres V–VI long, approximately 3.0 times longer than wide. Pronotum subrectangular, wider than long. Metafemur regularly swollen; ventral surface with three long stout bristles proximally and a row of 5–8 shorter bristles distally; metatibia hairy, with a prominent doubled apical spur (Figure 5I). Male abdominal tergite X convex posteromedially, without median projection; tergite XI with median upcurved projection; apex of male abdominal sternite VIII rounded. Male genitalia specific (Figure 5O), symmetrical, with two hook-like sclerites posteriorly; intromittent organ enlarged, connected to a heart-shaped, strongly sclerotized sclerite, from which it extends dorsally and posteriorly in conspicuous loop that leads to a membranous sac; basal plate composed of anterior tongue-like process and of posterior diverging lateral lobes [67].

Species included. *Cordezoros zimmermani* (Gurney, 1939), comb. nov.

Distribution. Oceanian region: Fiji (Figure 1C).

Etymology. The generic name refers to the heart-shaped sclerite of the male genitalia (corde in Latin), which is unique among Zoraptera, in combination with a suffix derived from the word base of the order name. Gender masculine.

Comments. *Cordezoros zimmermani* has unique male genitalia that differ markedly from those of all other described Zoraptera species [67]. The symmetrical genitalia, enlarged basal plate, hind tibia with only a pair of apical spurs, and presence of an intromittent organ well support the placement of this genus in the Spiralizoridae: Spiralizorinae. The phylogenetic position and detailed relationships of this taxon require further assessment by molecular methods, because the morphological homologization of genitalia is uncertain.

###### *Scapulizoros* gen. nov.

ZooBank: http://zoobank.org/urn:lsid:zoobank.org:act:C18F9E6A-5ECF-4A49-8EB4-8604B1A21527

Type species. *Zorotypus novobritannicus* Terry and Whiting, 2012; here designated.

Diagnosis. Body length 2.5–2.6 mm, basic color of apteron specimens pale to darker brown, alates and deälates dark brown. Antennae with nine antennomeres, antennomere I short, as long as or slightly longer than antennomere III, antennomere III 1.5 times longer than antennomere II; antennomeres V–VI long, 3.0 times longer than wide (Figure 1 in Terry and Whiting [68]). Pronotum subrectangular, wider than long. Metafemur swollen, ventral surface with 7 long stout bristles; metatibia with two apical spurs (Figure 5H). Male abdominal tergite X membranous medially, without median projection; tergite XI with small or long upcurved projection (mating hook) (Figure 13 in [68]); apex of male abdominal sternite VIII rounded. Male genitalia symmetrical, with large membranous area; basal plate M-shaped, posterior part protruding to strongly divergent lateral arms curved anteromedially near distal end; medial projection (intromittent organ?) emerging from posterior portion of the membranous area and curving around on the ventral surface of the genitalia (see Figures 14 and 15 in Terry and Whiting [68]).

Species included. *Scapulizoros novobritannicus* (Terry and Whiting, 2012), comb. nov.

Distribution. Oceanian region: Papua New Guinea (Figure 1C).

Etymology. The generic name refers to the unique lateral arms (scapulis in Latin) of the genitalia in combination with a suffix derived from the word base of the order name. Gender masculine.

Comments. *Scapulizoros novobritannicus* is sister to *Spiralizoros* as indicated in Figure 1 of the current study and in Matsumura et al. [5]. The species has unique male genitalia [68]. The symmetrical genitalia and hind tibiae with only one pair of apical spurs well support its placement in Spiralizorinae.

#### 3.2.3. Species Incertae Sedis in Zoraptera

We have not been able to assign the following nine species to the proper supraspecific rank:

##### *Zorotypus congensis* van Ryn Tournel, 1971

Comment. The description and illustration of the main diagnostic morphological characters of *Z. congensis* do not allow us to correctly assign this species to any supraspecific rank. Male genitalia are not described [69], but the presence of only two apical spurs on the hind tibiae does not agree with other African species of *Zorotypus* and suggests the placement of this species in Spiralizorinae or Spermozorinae. Since the type material is probably lost (Didier Van den Spiegel, Royal Museum for Central Africa, Tervuren, Belgium, pers. comm. 2015), the systematic position of *Z. congensis* remains unclear.

##### *Zorotypus javanicus* Silvestri, 1913

Comment. *Zorotypus javanicus* was described based on a nymph [11], and apterous adults were subsequently reported by Karny [58]. The taxonomically important morphological characters, however, have not been adequately described and illustrated, and this species, therefore, cannot be currently assigned to a proper supraspecific rank. The type depository is unknown.

##### *Zorotypus juninensis* Engel, 2000

Comment. Most of the diagnostic morphological characters of this species were not mentioned and/or illustrated in the original description [70], except for the antennae. The characters of the antennae suggest that this species might belong to Spiralizoridae: Spiralizorinae. Additional detailed morphological and molecular study is needed to determine the correct placement of this species. The type material is deposited in the American Museum of Natural History, New York.

##### *Zorotypus lawrencei* New, 1995

Comment. *Zorotypus lawrencei* was described based on two females, one apteron and one dealate [71]. Since the main taxonomical characters are based on the male genitalia, we must postpone the systematic placement of this species until a male is discovered. According to the spur composition on the metatibiae [71], *Z. lawrencei* probably belongs either to Zorotypidae: Spermozorinae or Spiralizoridae: Spiralizorinae. The type material is deposited in the Australian National Insect Collection, Canberra.

##### *Zorotypus leleupi* Weidner, 1976

Comment. *Zorotypus leleupi* is known only from type material composed of two females [72]. Since the main taxonomical characters are based on male genitalia, classification will not be possible until a male is described or molecular data are obtained. The presence and number of spurs on the metatibiae were not described. The type material seems to be lost (Kai Schuette, ZMH, pers. comm. 2015; Didier Van den Spiegel, Royal Museum for Central Africa, Tervuren, Belgium, pers. comm. 2015).

##### *Zorotypus longicercatus* Caudell, 1927

Comment. *Zorotypus longicercatus* is known only from the original description [73] based on two nymphs, and it therefore cannot be classified. The determination of the taxonomic position of this species will require the description of the male or molecular comparison. The holotype is deposited in the Museum of Natural History, Smithsonian Institution, Washington, DC, USA.

##### *Zorotypus newi* (Chao and Chen, 2000)

*Formosozoros newi* Chao and Chen, 2000; synonymized by Engel and Grimaldi [15].

Comment. *Zorotypus newi* is known only from type material composed of five females [14]. The species is characterized by extremely long cerci and tarsi with a long first tarsus. Metatibiae bear one apical spur. Lengths of basal antennomeres suggest affinity to Spermozorinae subfam. nov. or Spiralizorinae subfam. nov. The taxonomic position of this species (genus) will require molecular comparison and the description of male genitalia. The holotype is deposited in the collection of Tunghai University, Taiwan.

##### *Zorotypus sechellensis* Zompro, 2005

Comment. *Zorotypus sechellensis* is inadequately described, i.e., the principal diagnostic characters were neither described nor illustrated. According to the description [74], the holotype male specimen should be deposited in the Royal Museum for Central Africa, Tervuren, Belgium, but such material is not in evidence there (Didier Van den Spiegel, pers. comm. 2015). The rest of the material deposited in the author’s collection seems also to be lost (O. Zompro, pers. comm. 2015).

##### *Zorotypus swezeyi* Caudell, 1922

Comment. *Zorotypus swezeyi* was described based on female specimens [75]. Since the main taxonomically important characters are based on male genitalia, the proper placement of this species is not possible until a male is described. The holotype is deposited in the Museum of Natural History, Smithsonian Institution, Washington, DC, USA.

### 3.3. Generic Identification Key (Based on Males)

Remark: At the current state of knowledge, the generic identification of females is complicated due to their more uniform morphology. See Table S3 for the preliminary generic identification key to the females of Zoraptera.

1. Genitalia symmetrical (Figure 4D,E and Figure 5L,M,O,P,R), with basal plate enlarged, larger than rest of genitalia; elongated intromittent organ usually present....................................................................................................................4
- Genitalia asymmetrical (Figure 2K,M and Figure 3E,G), without recognizable basal plate; elongated intromittent organ absent.......................................................................................................................2
2. Antennomeres II and III of similar length (Figure 2A); metatibia with three spurs, of which only one is located apically (Figure 2F,J); pronotum wider than long or as long as wide.................................................................................3
- Antennomere III about 1.5–2.0 times longer than antennomere II (Figure 3B); metatibia with two apical spurs only (Figure 3D); pronotum longer than wide. Oriental region *Spermozoros* gen. nov.
3. Ventral edge of metatibia with three stout spurs (Figure 2F,J); mating hook on abdominal tergite X well developed, prolonged. Neotropical/Afrotropical regions..........................................................................................*Zorotypus* Silvestri, 1913, stat. revid.
- Ventral edge of metatibia with three inconspicuous spurs (Figure 2H); mating hook absent, tergites X and XI with small median protuberance. Nearctic region...........................................................................*Usazoros* Kukalova-Peck and Peck, 1993, stat. restit.
4. Metatibia with two stout spurs on ventral surface, of which only one is located apically (Figure 4C); antennomere I approximately as long as antennomeres II and III combined (Figure 4A). Abdominal tergites X and XI with median projections. Panamanian and Neotropical regions............................................................*Latinozoros* Kukalova-Peck and Peck, 1993, stat. restit.
- Metatibia with two small apical spurs or spurs absent (Figure 5J,K); antennomere I approximately as long as antennomere III (Figure 5B); abdominal tergite X flat, abdominal tergite XI with median projection (Figure 5S,T)...........................................................................................................................5
5. Elongated intromittent organ present (Figure 5L,M,O,R)................................................................................................................6
- Elongated intromittent organ absent (Figure 5P). Neotropical region.............................................*Brazilozoros* Kukalova-Peck and Peck, 1993, stat. restit.
6. Intromittent organ coiled (Figure 5L,M,R)..........................................................7
- Intromittent organ dorsoventrally looped (Figure 5O). Oceanian region........................................................................................................................8
7. Basal plate of genitalia anteriorly strongly dilated, with diverging sides (Figure 5L–N). Oriental region............................................................*Spiralizoros* gen. nov.
- Basal plate of genitalia not dilated anteriorly, with sides converging anteriorly (Figure 5R). Nearctic-Panamanian-Neotropical regions..............................*Centrozoros* gen. nov.
8. Distal half of metafemur with row of short stout bristles on ventral surface (Figure 5I). Male genitalia with strongly sclerotized heart-shaped sclerite (Figure 5O). Papua New Guinea........................................................................................................................*Cordezoros* gen. nov.
- Distal half of metafemur with row of long stout bristles on ventral surface (Figure 5H). Male genitalia without heart-shaped sclerite. Fiji..............................*Scapulizoros* gen. nov.

## 4. Discussion

### 4.1. Phylogeny and Classification of Zoraptera

Zoraptera show an extreme uniformity in general body morphology [1,2]. This has lead to the persistance for >100 years of a conservative classification of extant Zoraptera with only a single nominotypical genus in a single family [2]. Some previous studies reported a discrepancy between the uniform external morphology of the group and remarkable differences in the male genital structures, which indicates the existence of deep infraordinal lineages [5,19,20,21,76]. Relationships within the order were rigorously tested only recently by Matsumura et al. [5] using a combination of two nuclear (18S, H3) and two mitochondrial (16S, 12S) markers. The resulting phylogeny showed a subdivision of Zoraptera into three major clades, which mostly agrees with our results.

Here, we used a different dataset (including almost half of the described zorapteran diversity) with partly different molecular markers to reconstruct a phylogeny within Zoraptera. We also re-examined the morphological characters including male genitalia of most zorapteran species in order to assess the morphological support for the clades recovered by molecular analysis. Our results clearly revealed two major phylogenetic lineages, classified here as the families Zorotypidae and Spiralizoridae (Figure 1A). Each of these families was further divided into two statistically-supported subclades (subfamilies) with diagnostic characters on antennae, hind legs, abdominal tergites, and male genitalia. The most striking differences between the here-defined families Zorotypidae and Spiralizoridae are the structures of the male genitalia. Members of Zorotypidae have strongly asymmetrical male genitalia with two or three terminal sclerotized claspers, and without an elongated intromittent organ (Figure 2K,M and Figure 3E,G). Spiralizoridae, in contrast, have symmetrical male genitalia usually with an elongated intromittent organ and a well-developed basal plate (Figure 4D–F and Figure 5L–R).

As currently redefined, the family Zorotypidae includes the subfamily Zorotypinae with the genera *Zorotypus* and *Usazoros*, and the subfamily Spermozorinae with a single genus, *Spermozoros* (Figure 1A). Division of Zorotypidae into two subfamilies is robustly supported by both molecular phylogeny and morphology. Male genitalia of the Zorotypinae are large and have two strongly hooked claspers accompanied by small accessory sclerites (Figure 2K,M), while male genitalia of the Spermozorinae are small and reduced to three sclerites, the middle one being spatula-like and the aedeagus hooked or straight (Figure 3E,G). Both groups are also well defined by the number and position of metatibial spurs; Zorotypinae have metatibiae with three spurs (Figure 2F,H,J), while Spermozorinae have only one pair of reduced metatibial apical spurs (Figure 3D). Although our Zorotypidae fully agrees with Clade 1 of Matsumura et al. [5], the interrelationships within this clade differ between the studies. The analysis of Matsumura et al. [5] divided Zorotypidae into two subclades: the first with *Zorotypus hubbardi* (now in *Usazoros*) and *Z. impolitus* (now in *Spermozoros*), and the second with three *Zorotypus* species from the Afrotropical and Neotropical regions. However, the authors did not discuss the surprising relationship of *U. hubbardi* and *S. impolitus*. *Usazoros hubbardi* shares diagnostic characters of antennae, metatibiae, and male genitalia with the true *Zorotypus* as defined here. Since our finding that *Zorotypus* + *Usazoros* are sister to *Spermozoros* was highly statistically supported and because these clades are also robustly supported by morphology, we classify them here as two subfamilies. The genus *Spermozoros* (Spermozorinae) is composed of species with two different types of male genitalia. Genitalia of type A (with a hooked and strongly sclerotized aedeagus) (Figure 3E) are shared by *S. asymmetricus*, *S. medoensis*, and *S. sinensis*, while genitalia of type B (composed of three small, straight, and weakly sclerotized sclerites) (Figure 3G) are shared by *S. weiweii*, *S. impolitus*, and *S. huangi* [54,55,56,57]. *Spermozoros weiweii* and *S. impolitus* form a monophylum in all analyses, but the relationships between *S. asymmetricus* and *S. medoensis* (and their relationships with the clade *S. weiweii* + *S. impolitus*) remain unresolved (Figure 1A). These two groups might represent two genera, but because this hypothesis lacks substantial support, we have assigned all of the above-mentioned species to one genus until additional species are available for analysis.

The family Spiralizoridae includes the subfamilies Spiralizorinae (containing the genera *Centrozoros*, *Scapulizoros*, *Spiralizoros*, *Brazilozoros*, and *Cordezoros*) and Latinozorinae (*Latinozoros*), which fully correspond with clades 2 and 3 of Matsumura et al. [5]. The apparent apomorphies of the male genitalia and metatibiae justify the erection of these subfamilies. Male genitalia of Latinozorinae have a typical membranous anterior process (basal plate) that apparently underlies the elongated intromittent organ which encircles the membranous process during rest (Figure 4D–F). Spiralizorinae have genitalia that lie ventrally on an enlarged basal plate (Figure 5L–R), while the intromittent organ (if present) is usually coiled. Regarding the differences in metatibia, Latinozorinae have two stout spurs along the distal third and near the apex (Figure 4C), while Spiralizorinae have only two apical spurs or are without spurs (Figure 5J,K). The results of our molecular analyses fully agree with Matsumura et al. [5] in recovering the same clades, which we have defined as the genera *Brazilozoros*, *Centrozoros*, and *Scapulizoros* + *Spiralizoros*. The interrelationships among these clades, however, slightly differ between the two studies. Matsumura et al. [5] recovered *Brazilozoros* sister to the remaining genera, while our analyses placed *Brazilozoros* in a clade with *Centrozoros* (Figure 1A).

Our proposed classification is based mainly on a molecular phylogenetic analysis in combination with morphological characters of male genitalia. Unfortunately, researchers described some species based solely on immature or female specimens [11,64,71,73] or provided insufficient information on the male genitalia [52,62,70,74]. These species could not be properly assigned to supraspecific rank and therefore should remain incertae sedis (Table 2) until males are described or molecular phylogenetic study is conducted.

The reliable placement of fossil taxa is also complicated. Several external morphological characters such as the shape of antennae and metatibia allow the classification of males to a subfamily level, but the proper assignment of fossils to genera requires extensive morphological comparisons. The classification of fossil Zoraptera based on the critical evaluation of available morphological characters should be a focus of further research. The fossil taxa *Xenozorotypus* and *Octozoros* with apomorphic characters not present in extant genera [16,17,77,78,79,80,81] are not in conflict with our proposed classification and do not influence its stability. In addition, the currently defined *Octozoros* is apparently a “waste basket” for species from several unrelated lineages.

### 4.2. Reproduction Strategies in Zorotypidae and Spiralizoridae

The morphological differences in the basic structural plan of male genitalia clearly reflect different reproductive strategies in Zoraptera. Dallai et al. [82] reported striking differences in the reproductive strategies of *Spiralizoros magnicaudelli* (Spiralizoridae) and *Spermozoros impolitus* (Zorotypidae). Males of *S. magnicaudelli* transfer sperms by internal insemination during copulation, while males of *S. impolitus* deposit a spermatophore (containing only one giant sperm) externally on the tip of the female abdomen during the brief contact between the male and female abdomens [82]. Copulation with internal sperm transfer observed in *S. magnicaudelli* [82] and in *S. caudelli* [76] is quite similar to what was described earlier in the spiralizorides *Centrozoros gurneyi* [83] and *Latinozoros barberi* [84,85]. On the other hand, Gurney [50] and Shetlar [86] observed cases of brief copulation in *Usazoros hubbardi,* which belongs to Zorotypidae. Mating behavior of this species is not well described, but sperm is transmitted internally during copulation via a spermatophore [82]. A study of sperm structure in *Zorotypus shannoni* suggests an evolutionary trend shared with *U. hubbardi* and *S. impolitus* [20], and thus supports the inclusion of these genera in the Zorotypidae. On the other hand, *Spiralizoros magnicaudelli*, *S. caudelli*, *Centrozoros huxleyi*, and *Brazilozoros weidneri* share the similar sperm with the characteristically modified axonemes, which supports their placement in the Spiralizoridae. The possession of a long coiled intromittent organ, which is used for removing sperm from spermatheca [76], and the production of a large spermatophore represent two different strategies for reducing sperm competition. The elongated intromittent organ of the Spiralizoridae can apparently remove sperm deposited by a preceding copulation (a polyandric strategy), while the spermatophore of the Zorotypidae prevents a subsequent copulation, because the spermathecal sac would already be filled by a large spermatophore (a monandric strategy) [19].

Both the Spiralizorinae and Latinozorinae have a symmetrical type of male external genitalia, usually with an elongated intromittent organ, but the two groups may slightly differ in mating behavior [82]. In Spiralizorinae, each copulation lasts 15–30 min. and no secretes are transmitted during it due to absence of cephalic gland in male. In Latinozorinae, each copulation is brief (less than minute), copulations are repeated several times, and males have a cephalic gland (accompanied by a hairy patch on the vertex) that secretes a liquid that is imbibed by the female during copulation [84,85]. Similar hairy patches on the vertex occur (although the presence of a cephalic gland has not been confirmed) in some species of Zorotypidae (*Zorotypus shannoni, Z. amazoensis*, *Z. caxiuana, Z. asymmetristernum, Z. delamarei, Spermozoros impolitus,* and *S. weiweii*) and Spiralizorinae (*Brazilozoros huxleyi*), but the courtship feeding has not been studied/observed [5]. Dallai et al. [82] described in detail the courtship behaviour of *Spermozoros impolitus*, and did not report a nuptial gift.

### 4.3. Diversity and Distribution of Extant Zoraptera

The extant diversity of Zoraptera is much lower than for almost all other groups of Hexapoda, with only 44 extant species distributed mainly in the pan-tropical regions including Southeast Asia, Central and South America, Africa, as well as several oceanic islands such as Hawaii, Fiji, Samoa, and the Galapagos (Figure 1B,C) [1,2,87]. Only *Usazoros hubbardi* has expanded its range deeply into Nearctic region [88]. Zorapterans have not been recorded yet from the Australian region [2,89]. Researchers have suggested that the low diversity of Zoraptera might result from a poor collecting effort rather than from an actual paucity, and that the real diversity of this lineage could be highly underestimated [1,2,55]. Consistent with this hypothesis, molecular phylogenetic analyses suggest a high level of cryptic diversity in Zoraptera. Some traditionally-recognized species are, in fact, complexes of morphologically similar species. For example, *Latinozoros barberi* [62,63,87], which is widely distributed in the Panamanian and north neotropical regions, is a complex of species with similar external morphologies. The two undescribed *Latinozoros barberi*-like species examined in the current study clearly differ in their male genitalia. In this regard, the Spiralizorinae from Southeast Asia seem to be highly under-investigated because in our study of many populations from Borneo, we found at least two undescribed species of *Spiralizoros*. Similarly, Matsumura et al. [5] found striking genetic differences between different populations of both *Brazilozoros weidneri* and *B. huxleyi*, which indicates the existence of additional cryptic species.

Zorotypidae have a circumtropical distribution (Figure 1B). Zorotypinae have been found in Africa (Afrotropical and Madagascan regions) and America (Nearctic, Neotropical, and Panamanian regions), and Spermozorinae are restricted to the Oriental region. Within Spiralizoridae, the distribution of Latinozorinae is restricted to the Panamanian and north Neotropical regions, and Spiralizorinae are known from the tropics and subtropics of America, Asia, and Oceania, but not from Africa (Figure 1C). The current distribution of the main evolutionary lineages across all continents (except Australia) points to the Paleozoic origin of Zoraptera, which was confirmed by recent studies [3,4,5].

## 5. Conclusions

Our molecular phylogenetic analysis of Zoraptera revealed two major phylogenetic lineages with maximal statistical support. We classified these two lineages as families, and each could be divided into two robustly-supported subclades, i.e., subfamilies. The recognition of two families and four subfamilies is supported by synapomorphies in the structure and shape of male genitalia. Other taxonomically valuable characters included the number of spurs on metatibia and the lengths of the first three antennomeres. Although the general appearance and the external morphology of Zoraptera are unusually uniform, striking differences in the structure of male genitalia within the recovered monophyletic clades illustrate deep divergences of these old evolutionary lineages. We recognized and diagnosed nine genera of Zoraptera, and assigned 35 species to these genera based on morphological/molecular characters. Nine species remained unclassified due to a lack of adult males, inadequate description, or missing type material. Molecular analyses revealed a high level of cryptic diversity, with some traditionally recognized species being complexes of morphologically similar species.

## Figures and Tables

**Table 1:** List of taxa used for the phylogenetic reconstruction of Zoraptera, with voucher numbers, information on geographic origin, and GenBank accession numbers.

Order/Family	Genus/Species	Voucher Number	Geographic Origin	18S rRNA	16S rRNA	COI mtDNA
**Dermaptera**						
Forficulidae	*Forficula auricularia*	014	Czech Republic	MN790613	MN790594	MN790630
Forficulidae	*Anechura bipunctata*	065	Mongolia	MN790616	MN790596	MH853426 ^1^
Forficulidae	*Chelidurella acanthopygia*	002/016 *	Czech Republic	MN790612	MN790593	MH853444 ^1^
Anisolabididae	*Euborellia arcanum*	E8	USA	MN790614	MN790595	MH853422 ^1^
Diplatyidae	*Schizodiplatys* sp.	084	Brunei	MN790610	N/A	MN790628
Pyginicranidae	*Pyragropsis thoracica*	077	French Guiana	MN790611	N/A	MN790629
Pyginicranidae	*Cranopygia* sp.	081	Brunei	MN790615	N/A	MN790631
**Zoraptera**						
Zorotypidae	*Zorotypus delamarei*	71Z	Madagascar	MN790597	MN790583	MN790618
Zorotypidae	*Usazoros hubbardi*	Zohub	USA	DQ013288 ^2^	N/A	N/A
Zorotypidae	*Spermozoros asymmetricus*	4Z	Brunei	MN790598	MN790592	MN790617
Zorotypidae	*Spermozoros impolitus*	68Z	Malaysia	MN790601	N/A	N/A
Zorotypidae	*Spermozoros weiweii*	19Z	Brunei	MN790602	MN790591	MN790622
Zorotypidae	*Spermozoros medoensis*	-	China	KM246626 ^3^	JQ910991 ^3^	KJ467512 ^3^
Spiralizoridae	*Spiralizoros* sp. 1	KY330 (VN)	Vietnam	DQ013290 ^2^	N/A	N/A
Spiralizoridae	*Spiralizoros* sp. 2	KY325 (MY1)	Malaysia	DQ013289 ^2^	N/A	N/A
Spiralizoridae	*Spiralizoros caudelli*	44Z	Malaysia	MN790605	MN790585	MN790624
Spiralizoridae	*Spiralizoros magnicaudelli*	6Z	Malaysia	MN790600	MN790590	MN790627
Spiralizoridae	*Spiralizoros* sp. 3	13Z	Brunei	MN790608	MN790589	MN790623
Spiralizoridae	*Spiralizoros* sp. 4	8Z	Malaysia	MN790609	MN790587	MN790625
Spiralizoridae	*Spiralizoros cervicornis*	KY328 (MY2)	Malaysia	DQ013291 ^2^	N/A	N/A
Spiralizoridae	*Spiralizoros cervicornis*	1Z	Brunei	MN790599	MN790588	MN790626
Spiralizoridae	*Scapulizoros novobritannicus*	BYU ZO002	Papua New Guinea	AY521891 ^4^	EF623273 ^5^	N/A
Spiralizoridae	*Centrozoros* sp. 1	38Z	Ecuador	MN790607	MN790586	N/A
Spiralizoridae	*Centrozoros neotropicus*	43Z	Costa Rica	MN790606	MN790584	MN790619
Spiralizoridae	*Latinozoros* sp. 1	41Z	Dominican Republic	MN790603	N/A	MN790621
Spiralizoridae	*Latinozoros* sp. 2	48Z	French Guiana	MN790604	N/A	MN790620
Spiralizoridae	*Brazilozoros huxleyi*	-	-	JQ259055 ^6^	N/A	N/A
Spiralizoridae	*Brazilozoros weidneri*	-	-	JQ259056 ^6^	N/A	N/A

^1^ Kirstová et al. [29]; ^2^ Yoshizawa and Johnson [23]; ^3^ Ma et al. [24]; ^4^ Terry and Whiting [10]; ^5^ Terry and Whiting (unpublished); ^6^ Wang et al. [49]; * chimaeric taxon (isolate 002 for 18S rRNA, isolate 016 for 16S rRNA and COI mtDNA).

**Table 2:** Summary of updated suprageneric classification of Zoraptera.

Family	Subfamily	Genus	Species	Distribution
**Zorotypidae** Silvestri, 1913 stat. revid.	**Zorotypinae** Silvestri, 1913 stat. revid.	*Zorotypus* Silvestri, 1913, stat. revid.	*Zorotypus guineensis* Silvestri, 1913 *	Guinea, Ghana, Ivory Coast
			*Zorotypus asymmetristernum* Mashimo, 2019	Kenya
			*Zorotypus delamarei* Paulian, 1949	Madagascar
			*Zorotypus vinsoni* Paulian, 1951	Mauritius
			*Zorotypus shannoni* Gurney, 1938	Brazil
			*Zorotypus amazonensis* Rafael and Engel, 2006	Brazil
			*Zorotypus caxiuana* Rafael, Godoi and Engel, 2008	Brazil
		*Usazoros* Kukalova-Peck and Peck, 1993, stat. restit.	*Usazoros hubbardi* (Caudell, 1918), stat. restit. *	USA
	**Spermozorinae** subfam. nov.	*Spermozoros* gen. nov.	*Spermozoros asymmetricus* (Kočárek, 2017), comb. nov. *	Brunei
			*Spermozoros huangi* (Yin and Li, 2017), comb. nov.	China: Yunnan
			*Spermozoros impolitus* (Mashimo, Engel, Dallai, Beutel and Machida, 2013), comb. nov.	Peninsular Malaysia
			*Spermozoros medoensis* (Huang, 1976), comb. nov.	China: Tibet
			*Spermozoros sinensis* (Huang, 1974), comb. nov.	China: Tibet
			*Spermozoros weiweii* (Wang, Li and Cai, 2016), comb. nov.	Borneo
**Spiralizoridae** fam.nov.	**Spiralizorinae** subfam. nov.	*Spiralizoros* gen. nov.	*Spiralizoros cervicornis* (Mashimo, Yoshizawa and Engel, 2013), comb. nov. *	Peninsular Malaysia, Borneo
			*Spiralizoros buxtoni* (Karny, 1932), comb. nov.	Samoa
			*Spiralizoros caudelli* (Karny, 1922), comb. nov.	Peninsular Malaysia, Sumatra
			*Spiralizoros ceylonicus* (Silvestri, 1913), comb. nov.	Sri Lanka
			*Spiralizoros hainanensis* (Yin, Li and Wu, 2015), comb. nov.	China: Hainan
			*Spiralizoros magnicaudelli* (Mashimo, Engel, Dallai, Beutel and Machida, 2013), comb. nov.	Peninsular Malaysia, Borneo
			*Spiralizoros philippinensis* (Gurney, 1938), comb. nov.	Philippines
			*Spiralizoros silvestrii* (Karny, 1927), comb. nov.	Indonesia: Mentawai Islands
			*Spiralizoros* sp. 1	Vietnam [23]
			*Spiralizoros* sp. 2	Malaysia [23]
			*Spiralizoros* sp. 3	Brunei (unpublished)
			*Spiralizoros* sp. 4	Borneo (unpublished)
		*Centrozoros* Kukalova-Peck and Peck, 1993, stat. restit.	*Centrozoros gurneyi* (Choe, 1989), stat. restit. *	Costa Rica, Panama
			*Centrozoros cramptoni* (Gurney, 1938), comb. nov.	Guatemala
			*Centrozoros snyderi* (Caudell, 1920), comb. nov.	USA; Jamaica
			*Centrozoros hamiltoni* (New, 1978), comb. nov.	Colombia, Barbados
			*Centrozoros manni* (Caudell, 1923) comb. nov.	Bolivia, Peru
			*Centrozoros mexicanus* (Bolívar y Pieltain, 1940), comb. nov.	Mexico
			*Centrozoros neotropicus* (Silvestri, 1916), comb. nov.	Costa Rica
			*Centrozoros* sp. 1	Ecuador (unpublished)
		*Brazilozoros* Kukalova-Peck and Peck, 1993, stat. restit.	*Brazilozoros brasiliensis* (Silvestri, 1946), stat. restit. *	Brazil
			*Brazilozoros weidneri* (New, 1978), comb. nov.	Brazil
			*Brazilozoros huxleyi* (Bolívar y Pieltain and Coronado, 1963), comb. nov.	Brazil, Peru, Guyana, Ecuador
		*Scapulizoros* gen. nov.	*Scapulizoros novobritannicus* (Terry and Whiting, 2012), comb. nov. *	Papua New Guinea
		*Cordezoros* gen. nov.	*Cordezoros zimmermani* (Gurney, 1939), comb. nov. *	Fiji
	**Latinozorinae** subfam. nov.	*Latinozoros* Kukalova-Peck and Peck, 1993, stat. restit.	*Latinozoros barberi* (Gurney, 1938), stat. restit. *	Panama, Costa Rica, Venezuela (?), Trinidad and Tobago (?), Puerto Rico (?)
			*Latinozoros* sp. 1	Dominican Republic (unpublished)
			*Latinozoros* sp. 2	French Guiana (unpublished)
**Zoraptera *incertae sedis***			*Zorotypus congensis* van Ryn Tournel, 1971	Congo
			*Zorotypus javanicus* Silvestri, 1913	Indonesia: Java
			*Zorotypus juninensis* Engel, 2000	Peru
			*Zorotypus lawrencei* New, 1995	Christmas Island
			*Zorotypus leleupi* Weidner, 1976	Galapagos
			*Zorotypus longicercatus* Caudell, 1927	Jamaica
			*Zorotypus newi* (Chao and Chen, 2000)	Taiwan
			*Zorotypus sechellensis* Zompro, 2005	Seychelles
			*Zorotypus swezeyi* Caudell, 1922	Hawaii

* Type species of the genus.

## Supplementary Materials

The following are available online at https://www.mdpi.com/2075-4450/11/1/51/s1, Figure S1: Molecular phylogenetic hypothesis for Zoraptera (full tree); Table S1: Primers and thermal profiles employed for PCR amplification of target sequences; Table S2: Results of the Xia’s nucleotide substitution saturation test in DAMBE; Table S3: Generic identification key to the females of Zoraptera.
