# Supplementary material for: Molecular Phylogeny and Infraordinal Classification of Zoraptera (Insecta)
Source: Insects. 2020 Jan 12;11(1):51. doi: 10.3390/insects11010051 (PMC7023341; doi:10.3390/insects11010051)
Supplement: Supplementary file 1 [file insects-11-00051-s001.zip › Table_S3.docx]

**Table S1.** Generic identification key to the females of Zoraptera. * The generic identification of females is not possible in case of the American Spiralizorinae.

1. Metatibia with three spurs on ventral surface, of which only one is located apically (Figure 2F,J) *2*

- Metatibia with two stout spurs on ventral surface, of which only one is located apically, or metatibia with two small apical spurs or spurs absent 3

2. Ventral edge of metatibia with three stout spurs (Figure 2F,J). Neotropical/Afrotropical regions

*Zorotypus* Silvestri, 1913, stat. revid.

- Ventral edge of metatibia with three inconspicuous spurs (Figure 2H). Nearctic region

*Usazoros* Kukalova-Peck & Peck, 1993, stat. restit.

3. Metatibia with two stout spurs on ventral surface, of which only one is located apically (Figure 4C) Panamanian and Neotropical regions *Latinozoros* Kukalova-Peck & Peck, 1993, stat. restit.

- Metatibia with two apical spurs or spurs absent (Figure 5J,K) 4

4. Oriental and Oceanian regions 5

- Panamanian and Neotropical regions

*Centrozoros* gen. nov./*Brazilozoros* Kukalova-Peck & Peck, 1993, stat. restit.*

5. Oriental region .6

- Oceanian region 7

6. Metafemur with stout bristles on ventral surface, at least 3 apical bristles arise from broad conical projection of metatibia (Figure 3C) *Spermozoros* gen. nov.

- Metafemur with slender bristles on ventral surface, broad conical projections of metatibia not developed (Figure 5E) *Spiralizoros* gen. nov.

7. Distal half of metafemur with row of short bristles on ventral surface (Figure 5I). Papua New Guinea *Cordezoros* gen. nov.

- Distal half of metafemur with row of long bristles on ventral surface (Figure 5H). Fiji

*Scapulizoros* gen. nov.
