# Supplementary figures and images for: Molecular Phylogeny and Infraordinal Classification of Zoraptera (Insecta)
Source: Insects. 2020 Jan 12;11(1):51. doi: 10.3390/insects11010051 (PMC7023341; doi:10.3390/insects11010051)

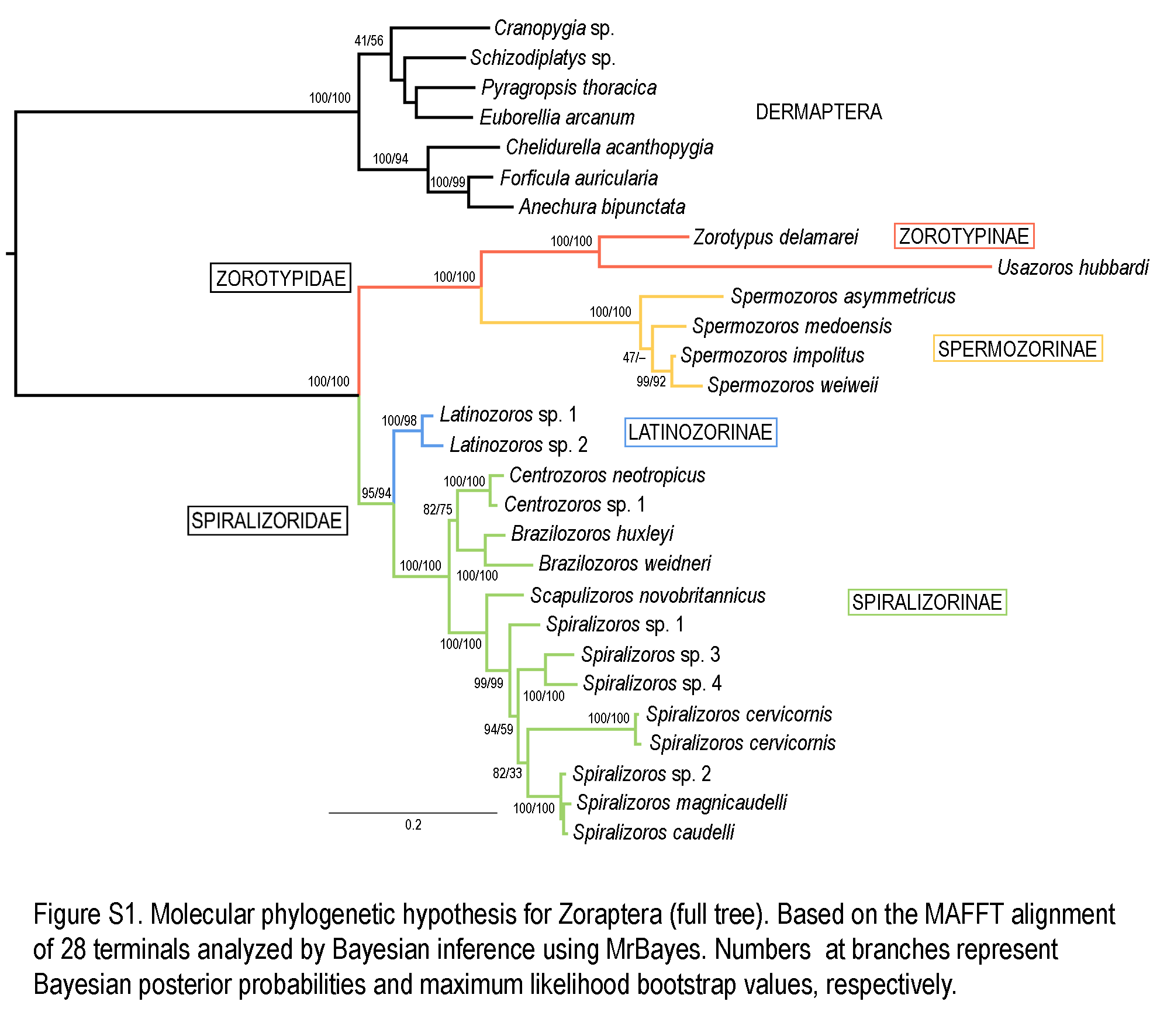

Supplement: Supplementary file 1 [file insects-11-00051-s001.zip › Figure_S1.tif]
