# Supplementary material for: Molecular Phylogeny and Infraordinal Classification of Zoraptera (Insecta)
Source: Insects. 2020 Jan 12;11(1):51. doi: 10.3390/insects11010051 (PMC7023341; doi:10.3390/insects11010051)
Supplement: Supplementary file 1 [file insects-11-00051-s001.zip › Table_S1.doc]

**Table S1.** Primers and thermal profiles employed for PCR amplification of target sequences.

| **Marker** | **Primer name** | **Primer sequence 5'→3'** | **PCR protocol** | **Primer reference** |
| --- | --- | --- | --- | --- |
| **16S** | 16Sar | CGCCTGTTTATCAAAAACAT | 3 min at 93 °C, 35x (30 s at 93 °C, 1 min at 48–53 °C, 90 s at 72 °C), 5 min at 72 °C | [33] |
|  | 16Sbr | CCGGTCTGAACTCAGATCACGT |  |  |
|  | 16S1471 | CCTGTTTANCAAAAACAT |  | [31] |
|  | 16S1472 | AGATAGAAACCAACCTGG |  |  |
| **18S**, 1^st^  fragment | 18S1F | CTGGTGCCAGCAGCCGCGGYAA | 2.5 min at 90 °C, 10x (50 s at 92 °C, 30 s at 45–53 °C, 1 min at 72 °C), 36x (30 s at 92 °C, 40 s at 45–53 °C, 1 min at 72 °C) 3 min at 72 °C | [34] |
|  | 18S2RC | TCCGTCAATTYCTTTAAGTT |  |  |
| **18S**, 2^nd^  fragment | 18Sa2.0 | ATGGTTGCAAAGCTGAAAC |  | [32] |
|  | 18S9R | GATCCTTCCGCAGGTTCACCTAC |  |  |
| **COI** | LCO1490 | GGTCAACAAATCATAAAGATATTGG | 2.5 min at 94 °C, 40x (30 s at 90 °C, 1 min at 49 °C, 1 min at 72 °C), 10 min at 72 °C | [30] |
|  | HCO2198 | TAAACTTCAGGGTGACCAAAAAATCA |  |  |
