# Supplementary material for: Molecular Phylogeny and Infraordinal Classification of Zoraptera (Insecta)
Source: Insects. 2020 Jan 12;11(1):51. doi: 10.3390/insects11010051 (PMC7023341; doi:10.3390/insects11010051)
Supplement: Supplementary file 1 [file insects-11-00051-s001.zip › Table_S2.doc]

Table S2. Results of the Xia's nucleotide substitution saturation test in DAMBE. Analysis performed on fully resolved sites only. Iss - index of substitution saturation; Iss.c^S^ - critical value for symmetrical tree topology; Iss.c^A^ - critical value for extremely asymmetrical tree topology; T - T value; DF - degrees of freedom; P^S^, P^A^ - probability that Iss is significantly different from the critical value (Iss.c^S^ or Iss.c^A^, respectively); Pinv - proportion of invariable sites.

Marker	/codon pos.	Iss	Iss.c^S^	T	DF	P^S^	Iss.c^A^	T	DF	P^A^	Pinv

18S rRNA	0.247	0.732	19.137	447	0.000	0.459	8.370	447	0.000	0.245

*rrnL* mtDNA	0.574	0.700	4.373	252	0.000	0.491	2.870	252	0.005	0.354

*cox1* mtDNA/1^st^	0.274	0.578	6.009	121	0.000	0.398	2.464	121	0.015	0.225

*cox1* mtDNA/2^nd^	0.114	0.578	10.358	98	0.000	0.398	6.351	98	0.000	0.371

*cox1* mtDNA/3^rd^	0.767	0.576	5.603	155	0.000	0.398	10.825	155	0.000	0.000
